# Supplementary material for: Epidemiological Dynamics in Populations Structured by Neighbourhoods and Households
Source: Bull Math Biol. 2025 Feb 27;87(4):50. doi: 10.1007/s11538-025-01426-0 (PMC11868190; doi:10.1007/s11538-025-01426-0)
Supplement: Supplementary file 1 — (pdf 195 KB) [file 11538_2025_1426_MOESM1_ESM.pdf]

Supplementary Information

Abby Barlow, Ben Adams, Sarah Penington

January 2024

1 Closed form expressions for R_* for $n = 2, 3$

We consider only a single neighbourhood here. For households fixed at size 2, the expected number of infections within the primary household with a single initial infection is $1 + \beta/(\beta + \gamma)$. The expected cases an infectious individual produces outside of their own household is α/γ. Hence, the household reproduction number is simply

$$R_* = \left(1 + \frac{\beta}{\beta + \gamma}\right)\left(\frac{\alpha}{\gamma}\right).$$

For populations of households of size 3, the construction of R_* for a single neighbourhood becomes more complex:

$$\begin{aligned} R_* = \Bigg[& \frac{\gamma}{\beta + \gamma} + 2\frac{\beta}{\beta + \gamma}\frac{2\gamma}{\beta + 2\gamma}\frac{\gamma}{\beta/2 + \gamma} \\ & + 3\left(\frac{\beta}{\beta + \gamma}\frac{\beta}{\beta + 2\gamma} + \frac{\beta}{\beta + \gamma}\frac{2\gamma}{\beta + 2\gamma}\frac{\beta/2}{\beta/2 + \gamma}\right)\Bigg]\left(\frac{\alpha}{\gamma}\right). \end{aligned}$$

Here, the terms in square brackets represent the expected number of infections within the primary household. We calculate this by walking through each possible path of events within the household.

2 Closed form expressions for offspring distributions for $n = 1, 2$

For a single neighbourhood of households fixed to size 1, the probability mass function of secondary infections is

$$g(m) = \frac{\alpha^m \gamma}{(\alpha + \gamma)^{m+1}}.$$

This is simply the geometric distribution with parameter $\alpha/(\alpha + \gamma)$ [1].

Whereas for a single neighbourhood of households size 2, the probability mass function of secondary infections becomes

$$g(m) = \alpha^m \left[\frac{\gamma(\beta + \gamma)}{\beta}\frac{1}{(\alpha + \beta + \gamma)^{m+1}} + \gamma^2(m + 1)\frac{1}{(\alpha + \gamma)^{m+2}} - \frac{\gamma^2}{\beta}\frac{1}{(\alpha + \gamma)^{m+1}}\right].$$

3 Multi-type branching process approximation for geometric offspring distribution

The probability generating functions are [2]

$$G_1(p_1, p_2) = \frac{1}{1 + R_*^{11}(1 - p_1) + R_*^{12}(1 - p_2)},$$
$$G_2(p_1, p_2) = \frac{1}{1 + R_*^{21}(1 - p_1) + R_*^{22}(1 - p_2)}.$$

4 Extra figures

References

[1] Joshua V Ross, Thomas House, and Matt J Keeling. Calculation of disease dynamics in a population of households. *PLoS One*, 5(3):e9666, 2010.

[2] Alun L Lloyd, Ji Zhang, and A Morgan Root. Stochasticity and heterogeneity in host–vector models. *J R Soc Interface*, 4(16):851–863, 2007.

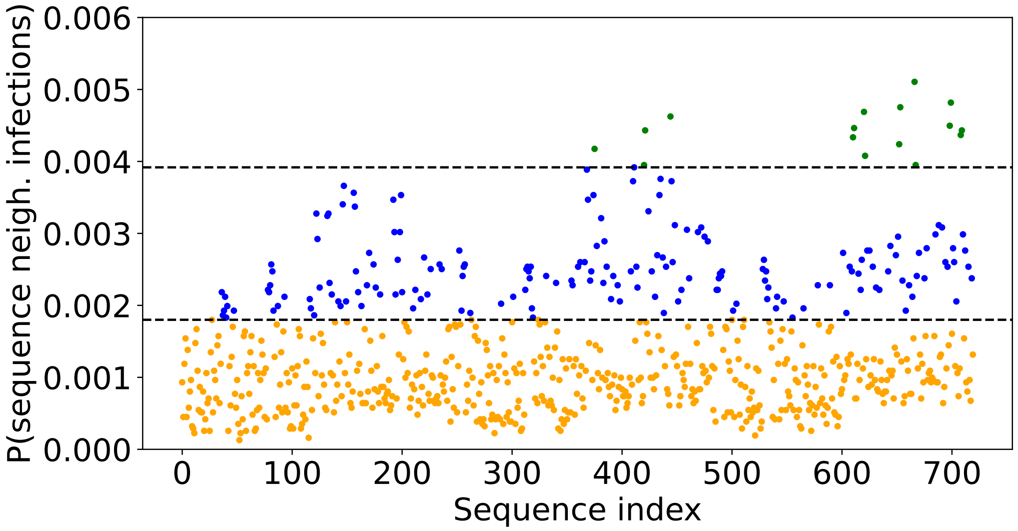

(a)

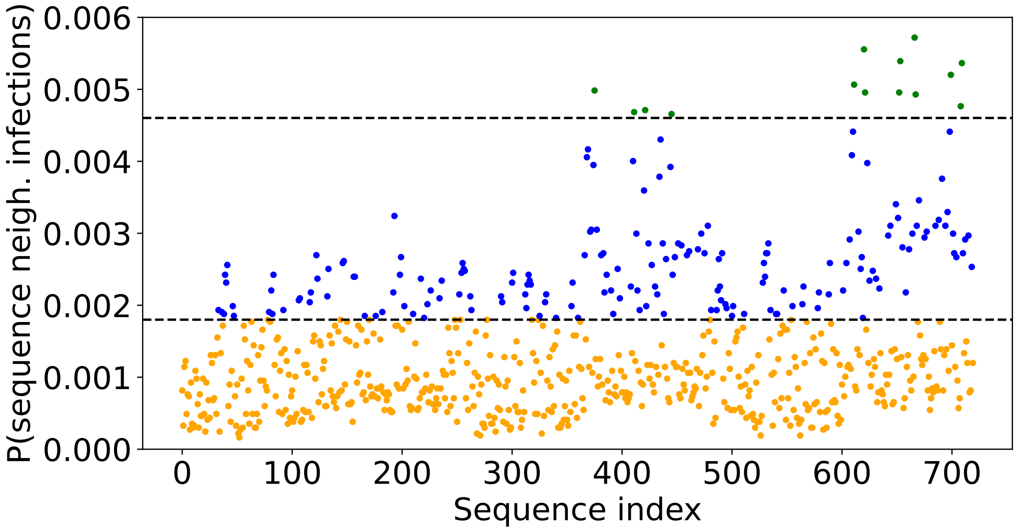

(b)

Figure 1: Probability an outbreak is observed through given sequences of neighbourhoods. There are 720 possible sequences of 6 neighbourhoods. Each point in the scatter plot corresponds to a unique sequence and shows the proportion of outbreaks in which the outbreak was observed in that sequence of neighbourhoods. Calculated from $50,000$ Gillespie SSA trials with those that did not result in significant outbreaks discarded. Initially a single individual was infected in a randomly chosen neighbourhood. $\nu = 3$ and r is assigned a value from the distribution $U[0, 0.1]$ (neighbourhoods 1,3,5) or $U[0.4, 0.5]$ (neighbourhoods 2,4,6). Clustering on Figure 9 in the main text was performed with Python using the 'sklearn.cluster' library and indicated by colour-coding the points. We show the same same cut-offs here for comparison. Points above the first dashed black line correspond to sequences that occurred in the top 6.9% of trials. Those above the second black line occurred in $\geq 0.18\%$ of outbreaks. (a) $\nu = 1$ and (b) $\nu = 5$.

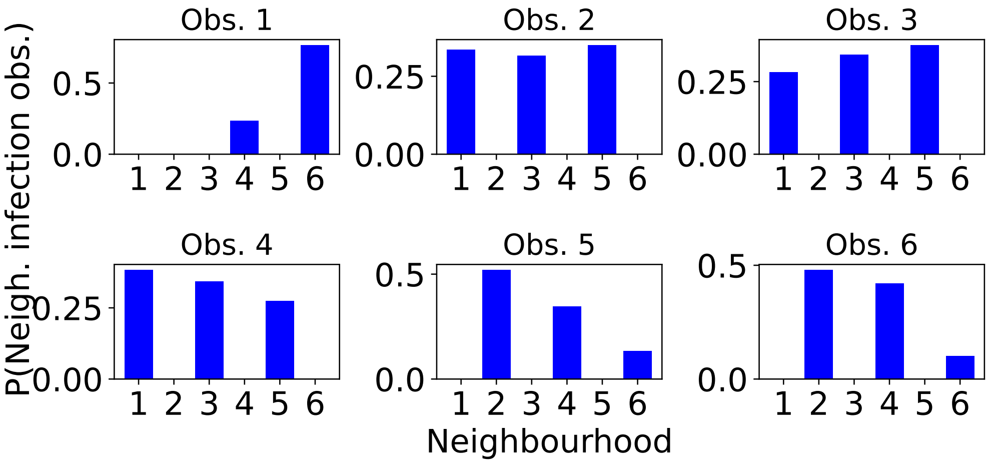

(a)

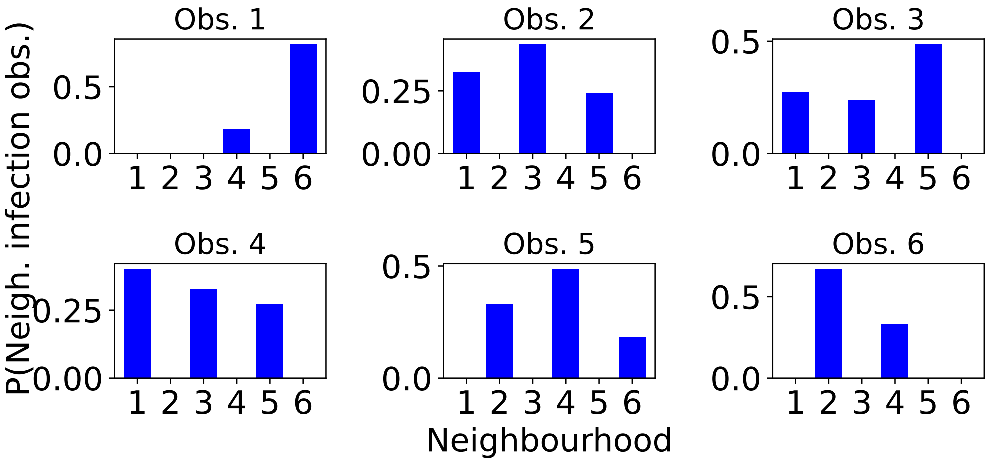

(b)

Figure 2: The probability a neighbourhood infection is observed in each neighbourhood. r takes value from either the distribution $U[0, 0.1]$ (neighbourhoods 1,3,5) or $U[0.4, 0.5]$ (neighbourhoods 2,4,6). $50,000$ realisations of Gillespie SSA were used with those not resulting in outbreaks being discarded. Of the remainder, only the top 6.9% of trials were used to construct this figure. From top left to bottom right the plots correspond to the 1st, 2nd, 3rd, …, 6th neighbourhood infection to be observed in the sequence of neighbourhood infections. (a) $\nu = 1$ and (b) $\nu = 5$.
